# Nitrogen Starvation Impacts the Photosynthetic Performance of *Porphyridium cruentum* as Revealed by Chlorophyll a Fluorescence

**DOI:** 10.1038/s41598-017-08428-6

**Published:** 2017-08-17

**Authors:** Long-Sheng Zhao, Kang Li, Qian-Min Wang, Xiao-Yan Song, Hai-Nan Su, Bin-Bin Xie, Xi-Ying Zhang, Feng Huang, Xiu-Lan Chen, Bai-Cheng Zhou, Yu-Zhong Zhang

**Affiliations:** 1 State Key Laboratory of Microbial Technology, Marine Biotechnology Research Center, Institute of Marine Science and Technology, Shandong University, Jinan, 250100 China; 2 Laboratory for Marine Biology and Biotechnology, Qingdao National Laboratory for Marine Science and Technology, Qingdao, China

## Abstract

Nitrogen is one of the most important nutrients needed for plants and algae to survive, and the photosynthetic ability of algae is related to nitrogen abundance. Red algae are unique photosynthetic eukaryotic organisms in the evolution of algae, as they contain phycobilisomes (PBSs) on their thylakoid membranes. In this report, the *in vivo* chlorophyll (Chl) a fluorescence kinetics of nitrogen-starved *Porphyridium cruentum* were analyzed to determine the effects of nitrogen deficiency on photosynthetic performance using a multi-color pulse amplitude modulation (PAM) chlorophyll fluorometer. Due to nitrogen starvation, the photochemical efficiency of PSII and the activity of PSII reaction centers (RCs) decreased, and photoinhibition of PSII occurred. The water-splitting system on the donor side of PSII was seriously impacted by nitrogen deficiency, leading to the inactivation of the oxygen-evolving complex (OEC) and decreased light energy conversion efficiency. In nitrogen-starved cells, a higher proportion of energy was used for photochemical reactions, and thermal dissipation was reduced, as shown by qP and qN. The ability of nitrogen-starved cells to tolerate and resist high photon flux densities was weakened. Our results showed that the photosynthetic performance of *P. cruentum* was severely impacted by nitrogen deficiency.

## Introduction

Nitrogen is an important nutrient for photosynthetic organisms. In a variety of ecosystems, including terrestrial and aquaculture environments, nitrogen source is a major determinant that limits the growth of green plants and algae. The response to nitrogen limitation varies among different microalgae. In many microalgae, lipid and polysaccharide accumulations as a carbon storage are often induced by nitrogen stress^1^. For example, both triacylglycerol and starch accumulate in the green alga *Chlamydomonas reinhardtii* ^2^ and red alga *Cyanidioschyzon merolae* ^3^.

Red algae are unique eukaryotic photosynthetic organisms, as they use giant protein complexes called phycobilisomes (PBSs) as their light-harvesting antennae on their thylakoid membranes, a primitive feature such as that found in cyanobacteria^4^. Light energy is trapped by the PBSs, and the energy is transferred to the reaction centers (RCs) of photosystems with high efficiency. PBSs are composed of phycobiliproteins and linker polypeptides. As the most abundant proteins in red algae and cyanobacteria, phycobiliproteins account for more than half of the total protein under optimum growth conditions.

In red algae and cyanobacteria, PBSs are degraded during nitrogen starvation. In the unicellular red alga *Porphyridium cruentum*, both the size of the PBSs and their density on thylakoid membranes were observed to be reduced during nitrogen starvation^5^. In addition, holes in thylakoid membranes were observed to increase in size during prolonged nitrogen-limited growth^5^. The degradation of PBSs not only provides a nitrogen source for cellular functions but also reduces the absorption capacity to prevent photodamage caused by overexcitation.

The growth rate of red algae decreases under nitrogen-limited growth conditions^6^. In addition to metabolic regulation, it was found that photosynthetic energy conversion efficiency is highly dependent on the supply of nitrogen in algae^7^. Chlorophyll (Chl) a fluorescence kinetics is closely related to photosynthesis of oxygen-evolving organisms^8^. This technique has been widely used to study the photosynthesis of red algae. Adaptations to different growth conditions have been studied, including irradiance^9, 10^, temperature^11, 12^, light quality^13^, heavy metal contents^14^, salinity^14, 15^, and CO_2_ levels^16^. Adaptations to tidal changes^17, 18^ and to seasonal and latitudinal variations^19^ have also been studied. However, research on the photosynthetic performance of red algae during nutrient limitation is relatively limited^20^.

In this work, we focused on the photosynthetic characteristics of the red alga *P. cruentum* to study Chl a fluorescence kinetics under nitrogen deficiency and discussed the influences of nitrogen deficiency on the photosynthesis and adaptation of red algae. We aim to better understand the survival mechanism of red algae in nitrogen-deficient environments.

## Results

### Chl concentration

The Chl content per cell decreased (Table 1), and the reduction reached 38.9% after 20 days of nitrogen-depleted cultivation compared to the Chl content in algal cells at 0 day. The reduction in Chl content was in accordance with the absorption spectra of Chl in *P. cruentum* cells in our previous study^5^. Each algal sample used in fluorescence kinetic experiments was diluted to a similar final Chl concentration (0.49–0.52 μg Chl ml^−1^). The densities of algal cells in each sample were measured, and the results are provided in Table 1.

**Table 1:** The Chl content of *P. cruentum* cells during nitrogen starvation, and the cell densities in each samples used in PAM measurement (n = 3).

Time (days)	Chl per cell ( × 10^−7^ μg)	Cell density ( × 10^6^ ml^−1^)
0	4.35 ± 0.31	1.20 ± 0.08
5	3.76 ± 0.25	1.31 ± 0.06
10	3.44 ± 0.17	1.51 ± 0.11
15	2.75 ± 0.08	1.80 ± 0.10
20	2.66 ± 0.15	1.87 ± 0.11

### Changes in slow Chl a fluorescence transients

**Figure 1:**
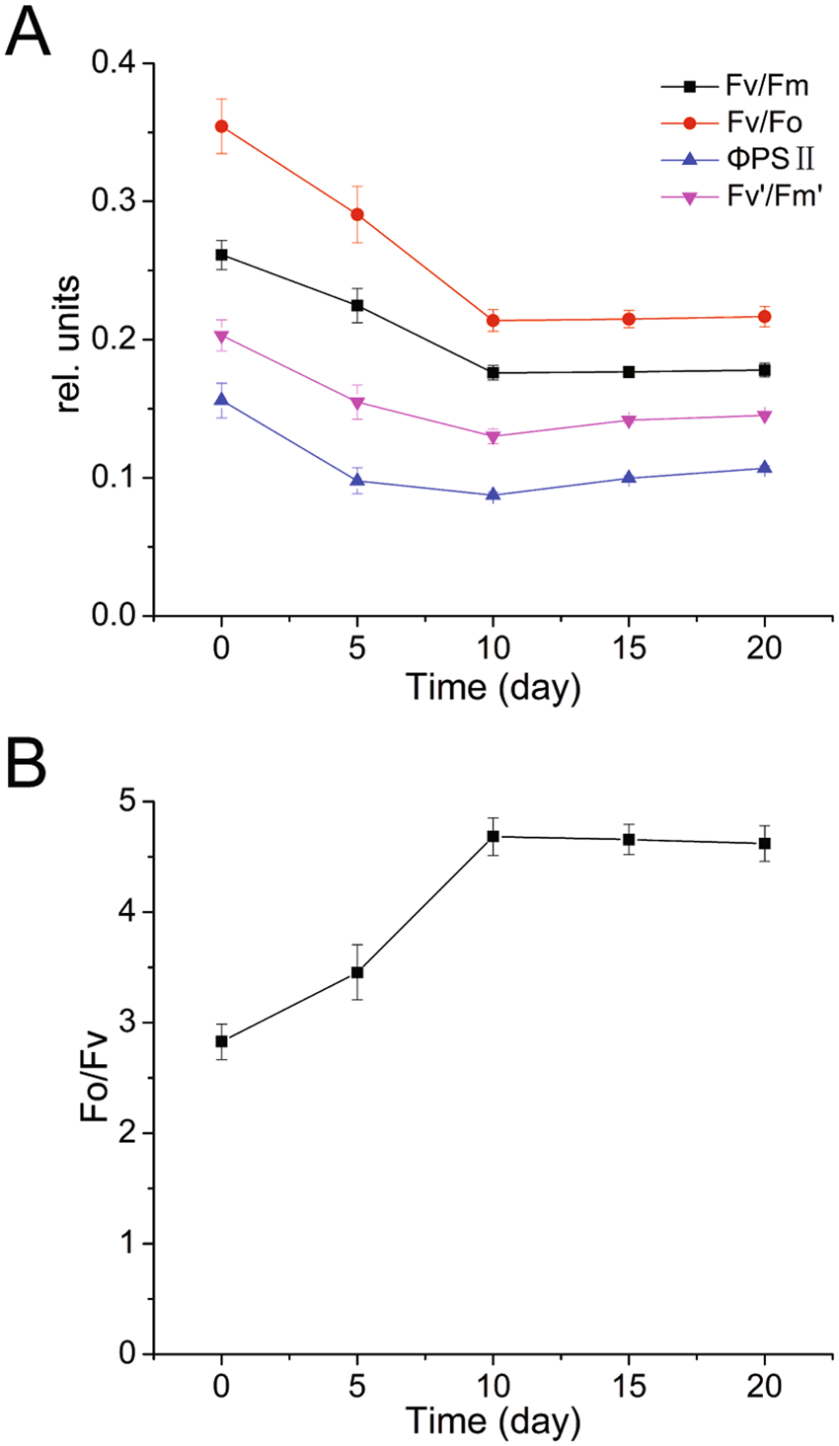
Changes in fluorescence parameters from slow Chl a fluorescence transients of nitrogen-starved *P. cruentum*. (**A**) Variation in the maximum photochemical efficiency of PSII (Fv/Fm), activity of PSII reaction centers (Fv/Fo), effective photochemical efficiency (ΦPSII), and light-adapted maximum photochemical efficiency of PSII (Fv’/Fm’) after nitrogen deficiency. (**B**) Variation in the efficiency of the OEC of PSII (Fo/Fv) after nitrogen deficiency.

The maximum photochemical efficiency of PSII (Fv/Fm) decreased approximately 32% during the initial period of the nitrogen deficiency and was stable after 10 days, which indicated that the light absorbed by the nitrogen-starved *P. cruentum* that was used in photosynthesis was reduced (Fig. 1A). The change in the active PSII reaction centers (Fv/Fo) was similar to that of Fv/Fm. Fv/Fo decreased by approximately 39% after 10 days and then stabilized along with nitrogen deficiency (Fig. 1A). The light-adapted maximum photochemical efficiency of PSII (Fv’/Fm’) decreased at first but then slightly increased after 10 days (Fig. 1A). The effective photochemical efficiency of PSII (ΦPSII) exhibited a similar tendency as did Fv’/Fm’ (Fig. 1A). The efficiency of the oxygen-evolving complex (OEC) of PSII, indicated by Fo/Fv, increased for 10 days and then stabilized (Fig. 1B).

**Figure 2:**
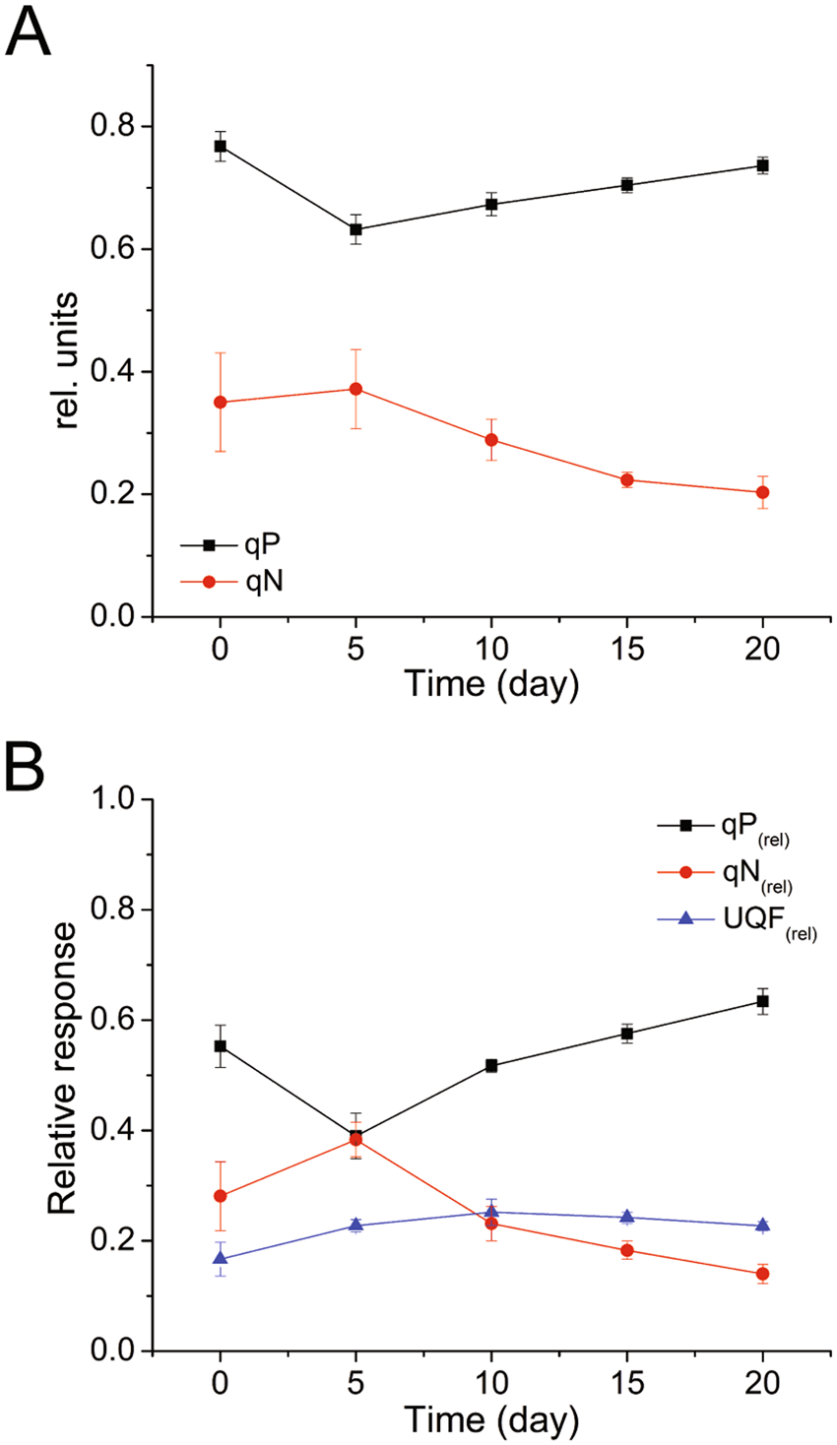
Distribution of energy dissipation of nitrogen-starved *P. cruentum*. (**A**) variation in the photochemical quenching coefficient (qP) and the non-photochemical quenching coefficient (qN) after nitrogen deficiency. (**B**) relative distribution of the dissipation energy processes through PSII of nitrogen-starved cells (qP_(rel)_, qN_(rel)_ and UQF_(rel)_).

The varied range of the photochemical quenching coefficient (qP) and non-photochemical quenching coefficient (qN) was not dramatic. As shown in Fig. 2A, qP decreased at first but then slowly increased; conversely, qN increased at first but then decreased slowly. This suggests that the proportion of opened RC was influenced by nitrogen deficiency. The change in qN indicated that thermal dissipation showed a downward trend. The degradation of phycobilin could affect the photosynthetic capacity of PSII. Along with the degradation of phycobilin, light absorption was reduced significantly, leading to an increase in photochemical efficiency and a decrease in thermal dissipation.

qP and qN were analyzed in a complementary manner by assessing the relative distribution of the energy dissipation processes through PSII (Fig. 2B)^21^. From 5 days to 20 days, qP_(rel)_ increased gradually; in contrast, qN_(rel)_ decreased gradually, which indicated that at later stages of nitrogen deficiency, most of the light energy trapped by PSII was used for photochemistry and that the non-photochemical quenching for energy dissipation was reduced. The proportion of UQF_(rel)_ tended to be stable.

### Rapid light curves and fit parameters

**Figure 3:**
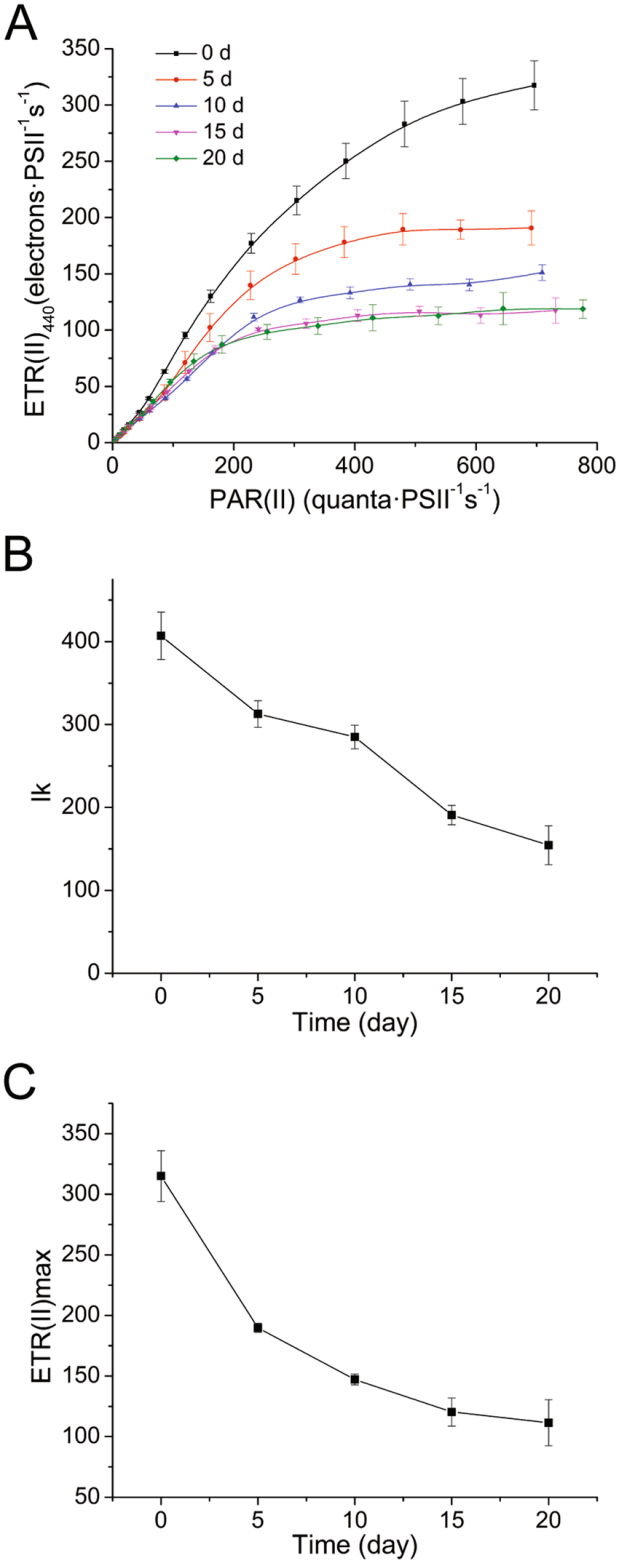
Variation in rapid light curves and fit parameters of nitrogen-starved *P. cruentum*. (**A**) ETR(II)-RLC of nitrogen-starved cells. (**B**) maximum electron transport rates ETR(II)_max_ fitted by EP model equations. (**C**) semi-light saturation point I_k_ fitted by EP model equations.

The effect of irradiance on photosynthesis is severely influenced by nitrogen deficiency in marine phytoplankton^7^. Our data showed that the light responses of PSII significantly decreased after nitrogen deficiency (Fig. 3A). The rate of relative photosynthetic electron transport decreased along with nitrogen-starving cultivation.

ETR(II) light curves were analyzed using EP model equations. The fit parameter I_k_ was the semi-light saturation point that reflected the resistance to high luminous intensity (Fig. 3B). The decreased I_k_ indicated that nitrogen deficiency reduced the capacity of cells to survive under high light intensity. The fit parameter ETR(II)_max_ decreased along with nitrogen deficiency (Fig. 3C), indicating that the maximum light-saturated photosynthetic capacity of nitrogen-starved cells was reduced.

### Polyphasic Chl a fluorescence transients

**Figure 4:**
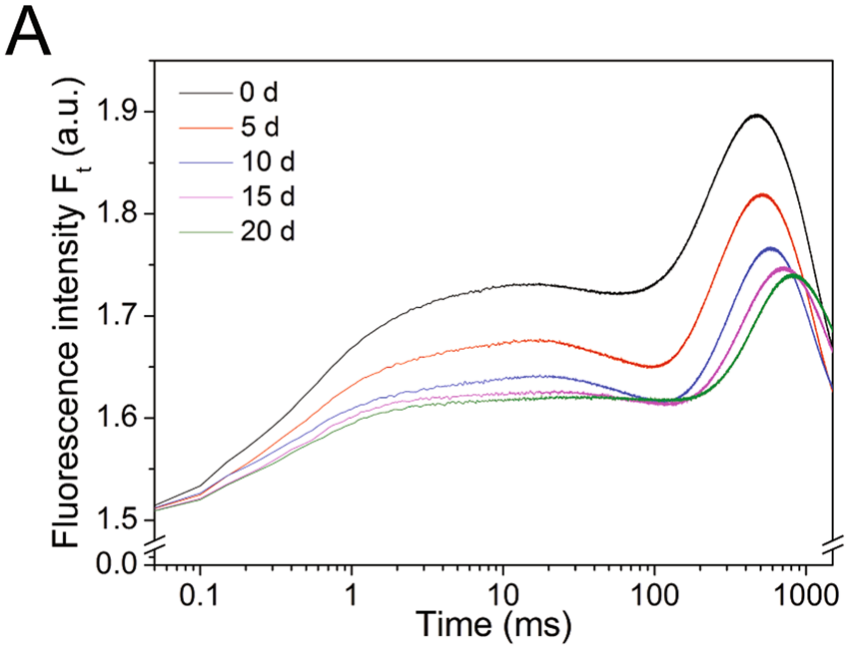
OJIP fluorescence transients of nitrogen-starved *P. cruentum* plotted on a logarithmic time scale.

A typical polyphasic O-J-I-P increase was evident in the curves (Fig. 4). Fluorescence transients in O-J, J-I and I-P phases were influenced by nitrogen starvation. The fluorescence transient in all phases decreased with cultivation time, whereas the time used to reach maximum fluorescence slightly increased along with nitrogen starvation (Fig. 4). Significant changes in fluorescence and different times needed to reach each intermediate step indicated that the photosynthetic units were affected by nitrogen deficiency.

**Figure 5:**
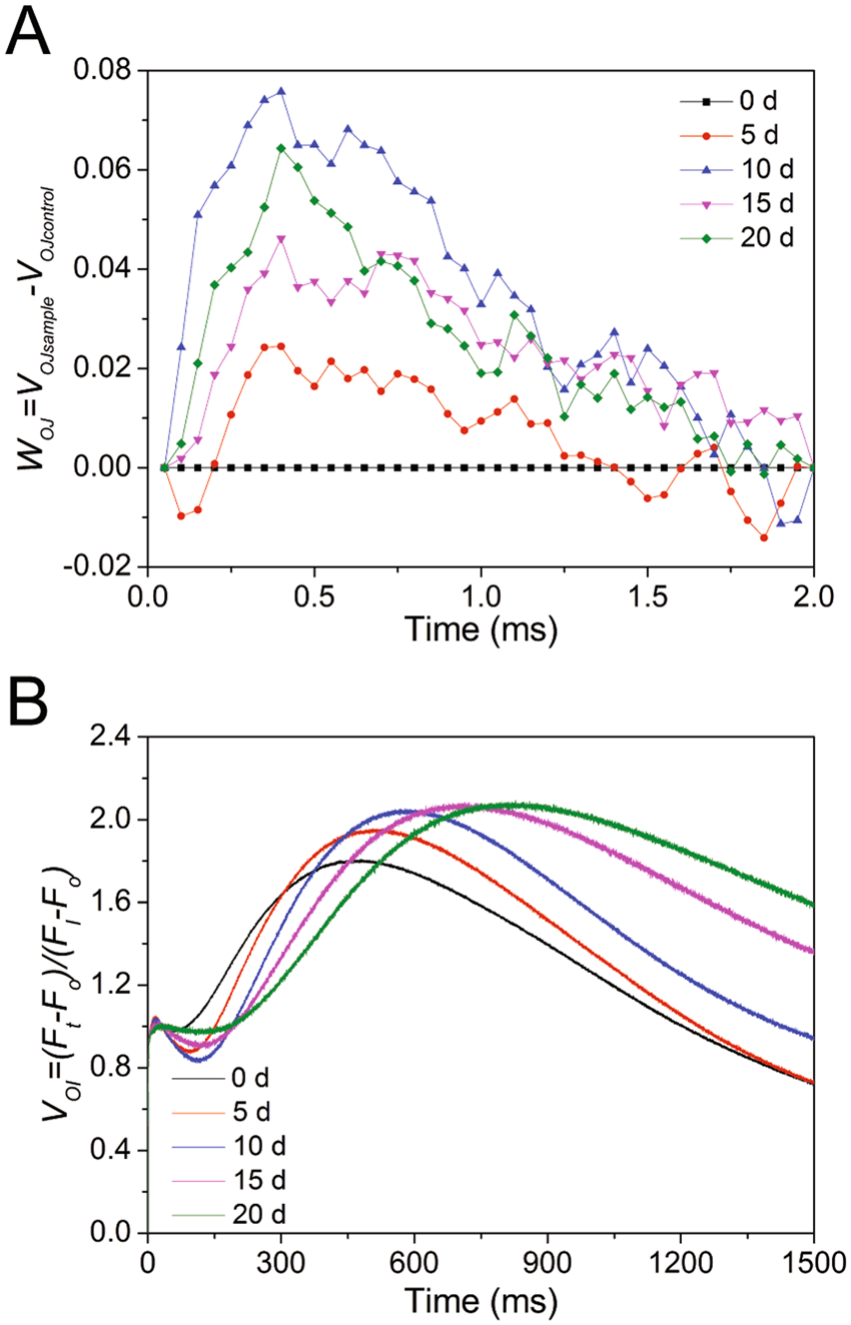
Fluorescence kinetics from the OJIP transients of nitrogen-starved *P. cruentum*. (**A**) kinetic differences in V_OJ_ obtained after double normalization between the steps O-J, showing the K-band at approximately 300 μs. (**B**) O-I normalized fluorescence transients.

Figure 5A shows the kinetic differences (W_OJ_ = V_OJsample_ − V_OJcontrol_) of V_OJ_ obtained after double normalization at the O-J phase, such that V_OJ_ = (F_t_ − F_o_)/(F_J_ − F_o_). W_OJ_ was positively influenced by nitrogen deficiency. The K-band was at approximately 300 μs and is related to the inactivation of the water-splitting system of the donor side of PSII^22^.

The pool size of terminal electron acceptor was analyzed by double normalization at the O and I phase, such that V_OI_ = (F_t_ − F_o_)/(F_I_ − F_o_) (Fig. 5B). The time to reach the P phase was influenced by nitrogen deficiency. The times were approximately 477, 514, 580, 705, and 813 ms for 0, 5, 10, 15, 20 days, respectively, indicating that nitrogen-starved *P. cruentum* needed more time to reach the P phase. The maximum amplitude of the transients increased gradually along with nitrogen deficiency, and from 10 days to 20 days, it was basically stable.

**Figure 6:**
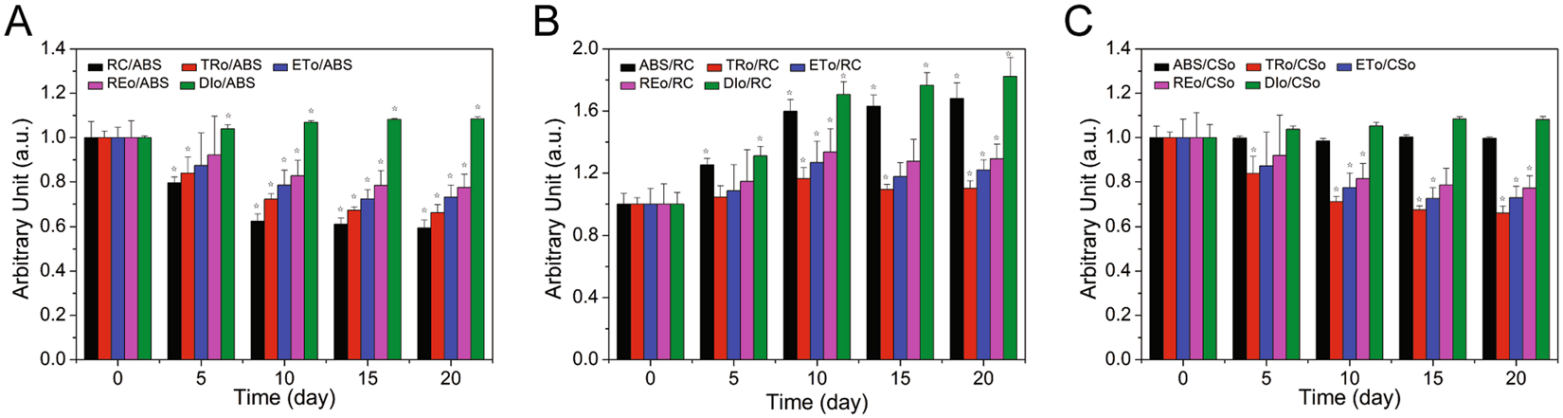
Photosynthetic parameters of nitrogen-starved fluorescence transients analyzed by the JIP test relative to those of cells at day 0. (**A**) energy flux ratios representing the following structural parameters: RC/ABS, density of PSII RC per absorption; TRo/ABS, maximum quantum yield for primary photochemistry; ETo/ABS, quantum yield for electron transport between the two photosystems; REo/ABS, the quantum yield for reducing terminal electron acceptors at PSI; and DIo/ABS, the quantum yield of energy dissipation. (**B**) specific energy fluxes (per active RC) representing the following functional parameters: ABS/RC, absorption flux; TRo/RC, trapping flux; ETo/RC, electron transport flux; REo/RC, electron flux for reducing terminal electron acceptors at the PSI side; and DIo/RC, dissipated energy flux. (**C**) the phenomenological energy fluxes (per excited cross-section (CS)), which are described as follows: ABS/CSo, absorption flux; TRo/CSo, trapped energy flux; ETo/CSo, electron transport flux; REo/CSo, electron flux for reducing terminal electron acceptors at the PSI side; and DIo/CSo, dissipated energy flux. (☆ = P < 0.05 vs. control)

The JIP test was used on the transients to analyze environmental effects on photosynthetic organisms. Figure 6A shows the structural parameter (energy flux ratios) changes resulting from nitrogen deficiency. ABS refers to the photon flux absorbed by the antenna pigments. The density of PSII RC per absorption (RC/ABS) decreased after nitrogen starvation. The TRo/ABS (maximum quantum yield) decreased during the initial period of the nitrogen deficiency and was stabilized after 10 days. The results suggested that the maximum photochemical efficiency was reduced in accordance with slow fluorescence induction kinetics. The ETo/ABS (quantum yield of electron transport between the two photosystems) and REo/ABS (quantum yield of reducing the terminal electron acceptor at PSI) had the same varying tendency as did TRo/ABS. The DIo/ABS (quantum yield of energy dissipation) changed weakly but was slightly promoted.

Specific energy fluxes were expressed per fully active PSII RC, called functional parameters (Fig. 6B). The ABS/RC (absorption flux per active RC) expresses antenna chlorophyll per active RC and increased after nitrogen starvation. The TRo/RC (trapped flux per active RC), ETo/RC (electron transport flux per active RC) and REo/RC (electron flux of reducing the terminal electron acceptor per active RC) of nitrogen-starved *P. cruentum* increased. The DIo/RC (dissipated energy flux per RC) showed a large increase after nitrogen starvation.

Figure 6C shows the phenomenological energy fluxes per excited cross-section (CS). The energy flux trapped per CS (TRo/CSo), the electron transport flux per CS (ETo/CSo), the electron flux of reducing the terminal electron acceptor per CS (REo/CSo) and the dissipated energy flux per CS (DIo/CSo) constituted the energy distribution of absorption flux per CS, according to the energy flux ratios. Due to the similarity with ABS/CSo, the changes in phenomenological energy fluxes were the same as with the energy flux ratios.

**Figure 7:**
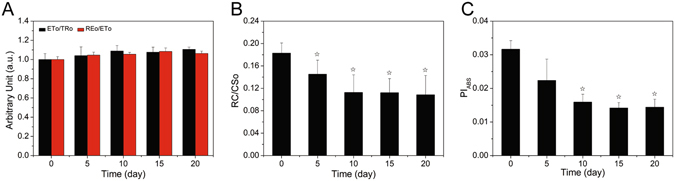
Photosynthetic parameters of nitrogen-starved fluorescence transients analyzed by the JIP test. (**A**) electron transport probabilities: ETo/TRo is the probability that a trapped exciton moves an electron into the electron transport chain beyond QA^−^, and REo/ETo is the probability that the intersystem electron carriers move to reduce the terminal electron acceptors at the PSI side. (**B**) density of active RCs of PSII per excited CS (RC/CSo). (**C**) summary of all partial forces represented by the single parameter performance index (PI). (☆ = P < 0.05 vs. control).

The probability for electron transport beyond QA^−^ (ETo/TRo) and the probability that the intersystem electron carriers move to reduce the terminal electron acceptor (REo/ETo) slightly increased in nitrogen-starved cells (Fig. 7A). The density of active RCs of PSII per excited CS (RC/CSo) decreased after nitrogen deficiency (Fig. 7B). The performance index was sensitive to nitrogen starvation, as shown in Fig. 7C. The PI_ABS_ of nitrogen-starved cells was significantly reduced.

### Reactive oxygen species (ROS) production

**Figure 8:**
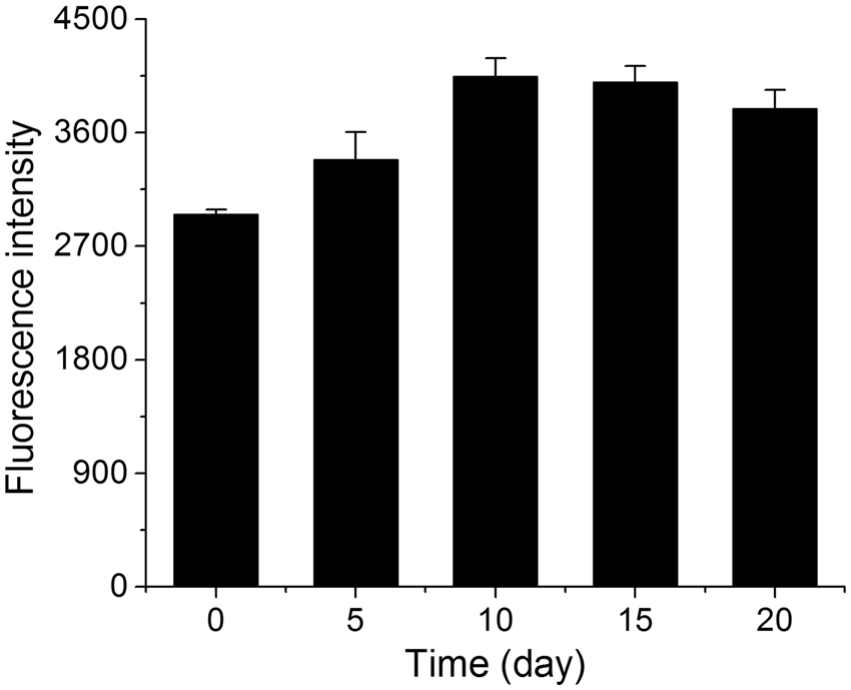
ROS production in *P. cruentum* cells during nitrogen starvation, represented by fluorescence intensity.

ROS production in *P. cruentum* cells during nitrogen starvation was measured. The results showed that the production of ROS was higher in algal cells during nitrogen starvation (from 5 to 20 days) compared with cells at 0 day (Fig. 8). ROS production decreased slightly from 10 to 20 days, which might be due to a decrease in Chl content and the degradation of photosynthetic membranes^5^.

## Discussion

Chl a and phycobilin contents decreased after nitrogen-depleted cultivation^5, 23^. These photosynthetic pigments are responsible for light harvesting and transferring absorbed light to the photosynthetic reaction centers^24^, and their concentration is related to photosynthetic efficiency^25^. The decrease in pigment contents could be one of the reasons for the decline of photosynthetic activity after nitrogen deficiency. The decrease in Fv/Fm represents the photoinhibition of PSII^26^, and our results showed that photoinhibition occurred in nitrogen-starved cells. The decrease in ΦPSII indicated that the energy used for electron transport was reduced. The activity of PSII reaction centers (Fv/Fo) of nitrogen-starved cells decreased, indicating that the photosynthetic apparatus was damaged and that light energy conversion efficiency decreased^27, 28^. The alteration of PSII photochemical reactions suggested that the ability of the photosynthetic apparatus to maintain QA in an oxidative state weakened.

The increase in Fo/Fv indicated that the water-splitting system of the donor side of PSII might be seriously impacted by nitrogen deficiency, leading to inactivation of the OEC; this could be confirmed by the positive K-band of the O-J phase. The damage to the OEC (inactivation of RCs) prevented overproduction of reduced QA^−^, which might be a plant response to avoid photoinhibition. Damage to the OEC is the primary step of the so-called two-step hypothesis to reveal the molecular mechanism of the primary photodamaging reaction^29, 30^, and the secondary damage occurred in the RC of PSII, leading to an increased lifetime of P680^+^ and the formation of singlet oxygen^30^. The inefficient reduction of P680^+^ resulted in a decrease in charge separation efficiency, as indicated by the Fv/Fm. Under nitrogen deficiency, the capacity for protein synthesis is reduced^7^, and the synthesis of the water-oxidizing enzyme system might be affected. As a result, the entire photosynthetic electron transport process between the photosystems might be altered, and the reoxidation of the PQ pool could be limited^21^. Our results showed that the ΦPSII, ETR(II), ETo/ABS, REo/ABS and the PQ pool size were affected. It was suggested that the low availability of terminal electron acceptors leads to a mostly reduced PQ pool under nitrogen-limiting conditions, which could induce acceptor-side PSII photoinhibition and the formation of ROS^31^. Our experiment confirmed that ROS production increased in *P. cruentum* cells during nitrogen starvation.

The energy distribution of photosynthesis after nitrogen deficiency was determined by qP and qN. It could be suggested that the variation in qP and qN were due to the degradation of PBSs and the variation in photochemical efficiency. The photosynthetic process was damaged after nitrogen starvation; however, the decrease in photochemical efficiency was mainly at the early stage when the degradation of PBSs were less serious^5^ and when the light-harvesting ability was maintained. To protect the photosystems from photodamage, non-photochemical quenching processes were activated to dissipate light energy. At 5 days, the descending degree of ΦPSII was larger than Fv/Fm; therefore, the proportion of the trapped energy used for photochemical quenching was reduced, and the proportion used for non-photochemical quenching increased. At the middle-late stages, the photochemical efficiency tended to be relatively stable, and the PBSs were severely degraded, which reduced the light-harvesting ability. The light energy transferred to PSII decreased, resulting in a higher proportion of energy being used for photochemical reactions; at the same time, the thermal dissipation decreased.

The values of qP_(rel)_, qN_(rel)_ and UQF_(rel)_ represent the relative distribution of the energy dissipation processes. The light energy that was not used in photochemistry was mostly dissipated by non-photochemical processes and unquenched fluorescence. At 5 days, the increased qN_(rel)_ revealed that a large amount of light energy was dissipated by non-photochemical processes. After 5 days, the energy used in photochemistry gradually increased, indicating the regulation of photoprotection after nitrogen deficiency. The UQF_(rel)_ represents the PSII reaction centers in the reduced state due to the limited reoxidation of QA. As such, the electron transfer between photosystems was impaired^32^.

The light responses of PSII were profoundly influenced by nitrogen deficiency. The light-saturated photosynthetic rates and semi-light saturation point decreased, indicating that the ability of nitrogen-starved cells to tolerate and resist high continuous photon flux densities was weakened. Thus, nitrogen-starved cells were more susceptible to photoinhibition. Variation in the activity of PSII RCs (Fv/Fo) and the density of active PSII RCs (RC/CSo) after nitrogen deficiency could be responsible for the increased sensitivity to photoinhibition.

The J and I phases were influenced by nitrogen deficiency. The variation in the J phase might be caused by change in the ability of electron transport beyond Q_A_^−^, and variation in the I phase might be caused by the ability of the intersystem electron carriers that move to reduce the terminal electron acceptor. When electron transport beyond Q_A_^−^ increased, less Q_A_^−^ accumulated, leading to a decrease in fluorescence during the J phase^33^. When the electron carriers moving to reduce the terminal electron acceptor increased, the accumulation of the reduced Q_A_ and PQ pools was reduced, leading to a decrease in fluorescence during the I phase^34^. The electron transport ability in PSII and between the photosystems increased after nitrogen deficiency, as shown by ETo/TRo and REo/ETo. Thus, nitrogen deficiency could reduce the light absorption and conversion through the degradation of photosynthetic pigments and damage to the OEC, but the ability of electron transport was not reduced.

It has been reported that the time for reaching the P phase is reduced along with the enhancement in light intensity^35^. Our results showed that t_Fm_ increased after nitrogen starvation, indicating that the rate of light trapping and electron transport was decreasing. The reduction of electron acceptors at PSI is shown by V_OI_. The maximum amplitude of the transients reflected the pool size of the terminal electron acceptor^33^. Our results suggested that the pool size of nitrogen-starved cells increased. In contrast, the pool size of nitrogen-starved rice decreased^33^.

The antenna chlorophyll per active RC (ABS/RC) increased after nitrogen deficiency. This might be caused by the decrease in active RCs. The increases in TRo/RC, ETo/RC, REo/RC and DIo/RC were also due to the reasons above. The decreases in TRo/CSo, ETo/CSo and REo/CSo indicated that the photosynthetic capacity per CS was reduced. The antenna chlorophyll energy dissipation of nitrogen-starved cells increased, as shown in our results (DIo/ABS, DIo/RC, DIo/CSo).

It has been reported that more than half of the nitrogen in a leaf is located in photosynthetic instruments, and photosynthesis is strongly affected by nitrogen availability^36^. However, some reports contrast with this view. The CO_2_ fixation rate of sunflower plants decreases under nitrogen deficiency, but the maximum quantum yield of primary photochemistry (Fv/Fm) is not reduced^37^. It was considered that the light reactions of photosynthesis of sunflower leaves are not influenced by nitrogen deficiency. It has been reported that low nutrient supply does not have a major effect on Fv/Fm or PI in *Graptophyllum reticulatum* ^38^. Our study showed that the photosynthetic performance decreased due to nitrogen deficiency.

## Methods

### Sample cultivation

The nitrogen-starving treatment of *P. cruentum* followed protocols in previous reports^5^. In brief, algal cells were first grown in artificial seawater (ASW) medium^39^. Cells that grew to the late logarithmic phase in ASW medium were collected by centrifugation (10 min at 5000 g, room temperature). The pellet was washed three times in ASW medium lacking potassium nitrate (-NASW) and was then suspended in -NASW medium at a ratio of 0.1 g (wet weight) of cells:1 ml of medium. Two milliliters of the suspension was added to 200 ml of -NASW medium and then cultured at 25 °C under continuous illumination (50 μmol quanta m^−2^ s^−1^) for the nitrogen-starving treatment. Cultures taken at 0, 5, 10, 15 and 20 days were collected, with the culture collected at 0 day serving as the control sample.

### Chl content measurement

Chl a was extracted from whole cells using N, N-dimethylformamide (DMF) in accordance with a previously described procedure^40^. In brief, 6 ml of culture was collected and rinsed twice in 0.5 M potassium phosphate (pH 7.0). The pellets were suspended in 1 ml of DMF and extracted at 4 °C for 3 hours in the dark. Then, the samples were centrifuged, and the concentration of Chl a in the supernatant extract was determined according to their absorption spectra^41^.

Algal cells in each sample were resuspended in -NASW medium to a similar final Chl concentration (0.49–0.52 μg Chl ml^−1^) for use in fluorescence kinetic experiments. The concentrations of algal cells were determined using the blood-counting chamber method.

### Slow Chl a fluorescence transient measurements

*In vivo* chlorophyll a fluorescence was measured using a multi-color pulse amplitude modulation (PAM) chlorophyll fluorometer (Heinz Walz, Effeltrich, Germany) connected to a PC and using PamWin software. During the measurements, samples were not stirred to avoid any bias in fluorescence signal due to movement of cells between dark and illuminated zones in the cuvette^21, 42^ and were kept at 25 °C using a thermostatic water bath. The treatments of samples were the same in the following experimental sections. Slow Chl a fluorescence transients were measured in saturation pulse (SP) analysis mode. Samples were kept in the dark for approximately 30 min in the presence of weak far-red (FR) background light to allow complete oxidation of PSII reaction centers before the measurements. The dark-adapted samples were induced by a measuring light (440 nm) to determine the minimum fluorescence yield (Fo) just prior to the SP. The maximum fluorescence yield (Fm) was induced by the first SP. The maximum photochemical efficiency of PSII (Fv/Fm = (Fm − Fo)/Fm) was then calculated^43^. The state of the water photooxidation process reflected by the efficiency of the OEC of PSII (Fo/Fv)^44^ and the activity of PSII reaction centers (Fv/Fo) were also calculated^45^.

Forty seconds after the first SP, an actinic light (440 nm) illuminated the samples, and a SP was applied every 20 s until the samples were completely light-adapted and the current fluorescence yield (Ft) and the maximum fluorescence yield (Fm’) in the light were stable; the stable Ft is referred to as the Fs. The effective photochemical efficiency of the light-adapted cells [ΦPSII = (Fm’ − Fs)/Fm’] was calculated^45^. The actinic light was turned off, and FR was applied to ensure the rapid and complete oxidation of the electron acceptors of PSII RCs after the samples were completely light adapted. The fluorescence yield (Fo’) at that moment was then measured. The maximum photochemical efficiency of PSII of light-adapted cells [Fv’/Fm’ = (Fm’ − Fo’)/Fm’] was then calculated.

The photochemical quenching coefficient qP [(Fm’ − Ft)/(Fm’ − Fo’)] represents the proportion of light energy trapped by open PSII RCs and used for electron transport. The non-photochemical quenching coefficient qN [1 − (Fm’ − Fo’)/(Fm − Fo)] reflects light energy dissipation not related to photochemistry and represents all the non-radiative processes of de-excitation^21, 46, 47^.

### Rapid light curve measurements

Rapid light curves (RLCs) were measured in SP analysis mode using the Light Curve Program files. The wavelength of light was 440 nm. The step width at each intensity setting was 1 min. Samples were dark adapted for 30 min in the presence of weak FR background light. The sample- and wavelength-dependent absorption cross-section of PSII, Sigma(II)_λ_, was estimated by the O-I_1_ increase curves recorded for the fast fluorescence transients^30^.

The ETR(II)-RLC represents the relationship of the rate of photosynthetic electron transport in PSII (ETR(II)) and the rate of quantum absorption of PSII (PAR(II)). The absolute rate of the photosynthetic electron transport of PSII was calculated as follows^30^:

1 $$\mathrm{ETR}(\mathrm{II})=\mathrm{PAR}(\mathrm{II})\cdot [{\rm{Y}}(\mathrm{II})/{{\rm{Y}}(\mathrm{II})}_{{\rm{\max }}}]$$

In Eq. (1), PAR(II) is the rate of quantum absorption of PSII, Y(II) is the effective PSII quantum yield ((Fm’–F)/Fm’), Y(II)_max_ is the PSII quantum yield (Fv/Fm) in the quasi-dark reference state, and ETR(II) is the rate of electron transport expressed in units of electrons per PSII per second. PAR(II) was calculated as follows:

2 $$\mathrm{PAR}(\mathrm{II})={\mathrm{Sigma}(\mathrm{II})}_{{\rm{\lambda }}}\cdot L\cdot {\rm{PAR}}$$

In Eq. (2), Sigma(II)_λ_ is the functional cross-section of PSII (nm^2^), *L* is Avogadro’s constant (mol^−1^), PAR is quantum flux density (or photon fluence rate), and PAR(II) is the rate of quantum absorption of PSII in units of quanta per PSII per second.

Light curves were fitted with EP model equations^48^. The fitting parameter ETR(II)max represents the maximum electron transport rates, and I_k_ is the semi-light saturation point.

### Fast Chl a fluorescence transients measurements

Fast Chl a fluorescence transients were measured in fast acquisition mode. Samples were dark adapted for approximately 30 min in the presence of a weak FR background light treatment. The fluorescence signal was digitized at 10-μs intervals. Chl a fluorescence transients were induced by a blue light having wavelength of 440 nm to generate maximum fluorescence intensity (Fm).

### JIP tests

Chl a transients were analyzed according to the equations of the JIP test^22, 49^. The fluorescence intensity at 50 μs was used as the Fo (O phase), and the fluorescence intensity at 300 μs (F_300μs_) was used to calculate the initial slope (Mo) of the variable component of the transient. The fluorescence intensity at 2 ms (F_J_) (J phase), the fluorescence intensity at 30 ms (F_I_) (I phase), and the maximum fluorescence intensity (Fm) (P phase) were used to calculate the parameters that quantify the energy flow through PSII^22, 34, 50^.

The flux ratios or yield expressed per absorption (ABS) were calculated, including the maximum quantum yield of PSII (φ_Po_ = TRo/ABS), the probability that a trapped exciton moves an electron into the electron transport chain beyond Q_A_^−^ (ψ_o_ = ETo/TRo), the quantum yield of electron transport (φ_Eo_ = ETo/ABS), the probability that the intersystem electron carriers move to reduce the terminal electron acceptor at the PSI (δ_Ro_ = REo/ETo), the quantum yield of reducing the terminal electron acceptor at the PSI (φ_Ro_ = REo/ABS)^33^, and the quantum yield of energy dissipation (φ_Do_ = 1 − φ_Po_). The fraction of active RCs of PSII per total absorption (RC/ABS) was also calculated.

Specific energy fluxes (per Q_A_^−^ reducing PSII RCs) were analyzed, which represent the energy distribution through PSII at the RC level, including the absorption flux (ABS/RC), trapped energy flux (TRo/RC), electron transport flux (ETo/RC), electron flux for reducing terminal electron acceptors at the PSI side (REo/RC)^33^, and dissipated energy at the level of the antenna chlorophylls (DIo/RC).

Phenomenological energy fluxes (per excited cross-section (CS)) were calculated, including the absorption flux (ABS/CSo), trapped energy flux (TRo/CSo), electron transport flux (ETo/CSo), electron flux for reducing terminal electron acceptors at the PSI side (REo/CSo), and dissipated energy flux (DIo/CSo). The density of active RCs of PSII per excited CS (RC/CSo) was also calculated.

The performance index on an absorption basis (PI_ABS_) that was used to estimate the initial stage of photosynthetic activity of a RC complex is regulated by three functional steps, namely, the absorption of light energy (ABS), trapping of excitation energy (TR) and conversion of excitation energy to electron transport (ET), expressed as follows:

3 $${{\rm{PI}}}_{{\rm{ABS}}}=[{{\rm{\gamma }}}_{{\rm{RC}}}/(1-{{\rm{\gamma }}}_{{\rm{RC}}})]\,\cdot \,[{{\rm{\phi }}}_{{\rm{Po}}}/(1-{{\rm{\phi }}}_{Po})]\,\cdot \,[{{\rm{\psi }}}_{{\rm{o}}}/(1-{{\rm{\psi }}}_{{\rm{o}}})]$$

In Eq. (3), γ_RC_ is the fraction of RC Chl (Chl_RC_) per total Chl (Chl_RC_ + Chl_Antenna_). The active RC density on a Chl basis can be represented as γ_RC_/(1 − γ_RC_) = Chl_RC_/Chl_Antenna_ = RC/ABS = [(F_J_ − F_o_)/4(F_300μs_ − F_o_)]·(Fv/Fm). The factor of 4 is used to express the initial fluorescence increase per millisecond^38, 51^. The expression φ_Po_/(1 − φ_Po_) = TRo/DIo = Fv/Fo estimated according to the JIP test is the contribution of the light reactions to primary photochemistry, which represents the performance due to the trapping probability (PTR). The contribution of electron transport beyond QA^−^ was calculated as ψ_o_/(1 − ψ_o_) = ETo/(TRo-ETo) = (Fm − F_J_)/(F_J_ − Fo).

Each of the abovementioned biophysical parameters was calculated from the original fluorescence measurements according to the formulas in Supplementary Table S1 ^38, 49, 51^. Statistical significances were determined using Student’s t-test.

### ROS production

The ROS production of algal cells was measured using 5-(and-6)-chloromethyl-2′,7′-dichlorodihydrofluorescein-diacetate, acetyl ester (CM-H_2_DCFH-DA)^52^. Cells (1 × 10^6^) were collected and rinsed in 0.5 M potassium phosphate (pH 7.0) by centrifugation. The pellet was suspended in 1 ml of 0.5% (v/v) CM-H_2_DCFH-DA and incubated at 37 °C for 20 min in the dark. Then, the sample was centrifuged and rinsed. The pellet was suspended in 400 μl of 0.5 M potassium phosphate and detected immediately using an EnSpire multimode plate reader (PerkinElmer, USA) with an excitation wavelength at 490 nm. ROS production was estimated by monitoring fluorescence at an emission wavelength of 530 nm.

## Electronic supplementary material

Supplementary information
