# Supplementary material for: Nitrogen Starvation Impacts the Photosynthetic Performance of Porphyridium cruentum as Revealed by Chlorophyll a Fluorescence
Source: Sci Rep. 2017 Aug 17;7:8542. doi: 10.1038/s41598-017-08428-6 (PMC5561210; doi:10.1038/s41598-017-08428-6)
Supplement: Supplementary file 1 — Supplementary information [file 41598_2017_8428_MOESM1_ESM.doc]

**Nitrogen Starvation Impacts the Photosynthetic Performance of *Porphyridium cruentum* as Revealed by Chlorophyll a Fluorescence**

Long-Sheng Zhao ^1^, Kang Li ^1^, Qian-Min Wang ^1^, Xiao-Yan Song ^1^,*, Hai-Nan Su ^1^,*, Bin-Bin Xie ^1^, Xi-Ying Zhang ^1^, Feng Huang ^1^, Xiu-Lan Chen ^1^, Bai-Cheng Zhou ^1^, Yu-Zhong Zhang ^1,2^

^1^ State Key Laboratory of Microbial Technology, Marine Biotechnology Research Center, Institute of Marine Science and Technology, Shandong University, Jinan 250100, China.

^2^ Laboratory for Marine Biology and Biotechnology, Qingdao National Laboratory for Marine Science and Technology, Qingdao, China.

^*^ **Corresponding author**: Hai-Nan Su. Tel: +86-531-88365013, Fax: +86-531-88564326, E-mail: suhn@sdu.edu.cn; Xiao-Yan Song. Tel: +86-531-88365013, Fax: +86-531-88564326, E-mail: xysong@sdu.edu.cn

**Supplementary Information**

**Supplementary Table 1.** Formulae and definitions of OJIP-test parameters

| Extract and technical parameters | | |
| --- | --- | --- |
| Fo = F_50μs_ | | fluorescence intensity at 50 μs |
| F_300μs_ | | fluorescence intensity at 300 μs |
| F_J_ | | fluorescence intensity at the J-step (at 2 ms) |
| F_I_ | | fluorescence intensity at the I-step (at 30 ms) |
| Fm | | maximal fluorescence intensity |
| t_Fm_ | | time to reach maximalFm |
| Vt = (Ft – Fo)/(Fm – Fo) | | relative variable fluorescence at time t |
| V_J_ = (F_J_ – Fo)/(Fm – Fo) | | relative variable fluorescence at J-step |
| V_I_ = (F_I_ – Fo)/(Fm – Fo) | | relative variable fluorescence at I-step |
| Mo = (dV/dt)o = 4 (F_300μs_ – Fo)/(Fm – Fo) | | initial slope of relative variable fluorescence |
| Yields or flux ratios | | |
| φ_Po_= TRo/ABS = 1 – (Fo/Fm) = Fv/Fm | | maximum quantum yield of PSII |
| ψ_o_= ETo/TRo = 1 – V_J_ | | probability that a trapped exciton moves an electronfurther than QA^-^ |
| φ_Eo_= ETo/ABS = Fv/Fm · (1 – V_J_) | | quantum yield of electron transport |
| δ_Ro_ = REo/ETo = (1 – V_I_)/(1 – V_J_) | | probability that the intersystem electron carriers move toreduce the end electron acceptors |
| φ_Ro_ = REo/ABS = Fv/Fm · (1 – V_I_) | | the quantum yield of reducing the end electron acceptors |
| φ_Do_ = 1 – φ_Po_ = (Fo/Fm) | | quantum yield of energy dissipation |
| Specific fluxes or specific activities | | |
| ABS/RC = Mo · (1/V_J_) · (1/φ_Po_) | | absorption flux per active RC |
| TRo/RC = Mo · (1/V_J_) | | trapping flux per active RC |
| ETo/RC = Mo · (1/V_J_) · (1 – V_J_) | | electron transport flux per active RC |
| REo/RC = Mo · (1/V_J_) · (1 – V_I_) | | electron flux for reducing end electronacceptorsper active RC |
| DIo/RC = (ABS/RC) – (TRo/RC) | | dissipation flux per active RC |
| Phenomenological fluxes or phenomenological activities | | |
| RC/CSo = Fv/Fm· (V_J_/Mo) · Fo | density of active reaction centers per excited CS | |
| ABS/CSo =Fo | absorption flux per excited CS | |
| TRo/CSo =φ_Po_ ·(ABS/CSo) | trapped flux per excited CS | |
| ETo/Cso=φ_Eo_ ·(ABS/CSo) | electron transport flux per excited CS | |
| REo/CSo = φ_Ro_·(ABS/CSo) | electron flux for reducing end electronacceptorsper excited CS | |
| DIo/CSo = (ABS/CSo) – (TRo/CSo) | dissipated flux per excited CS | |
| Performance index | | |
| PI_ABS_ = (γ_RC_/(1–γ_RC_)) · (φ_Po_/(1–φ_Po_)) · (ψ_o_/(1 –ψ_o_)) | performance index | |
| γ_RC_/(1–γ_RC_) = RC/ABS= [(F_J_ –Fo)/4(F_300μs_–Fo)]·(Fv/Fm) | RC-density on chlorophyll a basis | |
| φ_Po_/(1–φ_Po_) = TRo/DIo = Fv/Fo | flux ration trapping per dissipation | |
| ψ_o_/(1 –ψ_o_) = ETo/(TRo–ETo) = (Fm–F_J_)/(F_J_–Fo) | electron transport beyond QA^-^ | |
